# Assessment of genetic mutation frequency induced by oxidative stress in *Trypanosoma cruzi*

**DOI:** 10.1590/1678-4685-GMB-2017-0281

**Published:** 2018-06-11

**Authors:** Carolina Furtado Torres-Silva, Bruno Marçal Repolês, Hugo Oliveira Ornelas, Andréa Mara Macedo, Glória Regina Franco, Sérgio Danilo Junho Pena, Erich Birelli Tahara, Carlos Renato Machado

**Affiliations:** 1 Universidade Federal de Minas Gerais Universidade Federal de Minas Gerais Departamento de Bioquímica e Imunologia Belo HorizonteMG Brazil Departamento de Bioquímica e Imunologia, Universidade Federal de Minas Gerais, Belo Horizonte, MG, Brazil

**Keywords:** T. cruzi, DNA, mutation frequency, H_2_O_2_

## Abstract

*Trypanosoma cruzi* is the etiological agent of Chagas disease, a public health challenge due to its morbidity and mortality rates, which affects around 6-7 million people worldwide. Symptoms, response to chemotherapy, and the course of Chagas disease are greatly influenced by *T. cruzi*‘s intra-specific variability. Thus, DNA mutations in this parasite possibly play a key role in the wide range of clinical manifestations and in drug sensitivity. Indeed, the environmental conditions of oxidative stress faced by *T. cruzi* during its life cycle can generate genetic mutations. However, the lack of an established experimental design to assess mutation rates in *T. cruzi* precludes the study of conditions and mechanisms that potentially produce genomic variability in this parasite. We developed an assay that employs a reporter gene that, once mutated in specific positions, convert G418-sensitive into G418-insenstitive *T. cruzi*. We were able to determine the frequency of DNA mutations in *T. cruzi* exposed and non-exposed to oxidative insults assessing the number of colony-forming units in solid selective media after plating a defined number of cells. We verified that *T. cruzi*‘s spontaneous mutation frequency was comparable to those found in other eukaryotes, and that exposure to hydrogen peroxide promoted a two-fold increase in *T. cruzi*‘s mutation frequency. We hypothesize that genetic mutations in *T. cruzi* can arise from oxidative insults faced by this parasite during its life cycle.

## Introduction

*Trypanosoma cruzi* is the etiological agent of Chagas disease, a complex zoonosis that affects more than seventy genera of mammalian hosts (Zingales *et al.*, 2012; Baptista *et al.*, 2014). According to the World Health Organization (WHO), around 6-7 million people are affected by this disease in 21 countries, most of them in Latin America [WHO Chagas disease (American trypanosomiasis) fact sheet, 2017]. Also noteworthy is the fact that nowadays this disease is spreading to non-endemic regions due to human migration (Schmunis, 2007).

The life cycle of *T. cruzi* is complex and involves two hosts: an invertebrate and a mammalian. Humans are considered accidental hosts, in which the classic vectorial infection generally occurs at night when the blood-sucking triatomines defecate during feeding (Frasch, 2000). Once the feces droplets expelled by the triatomine reach the bloodstream or get in contact with eyes, nose or mouth mucosa, the infection is then perpetrated (Prata, 2001). Humans may also be infected with *T. cruzi* through blood transfusion, organ transplantation, from mother to infant during pregnancy, laboratory accidents, as well as through ingestion of food contaminated with triatomine feces (Shikanai-Yasuda *et al.*, 1991; de Noya and González, 2015).

Following the infection by *T. cruzi*, a short acute phase characterized by high parasitemia takes place, along with unspecific symptoms (Macedo *et al.*, 2004). During its chronic phase, Chagas disease presents a large spectrum of symptoms and low parasitemia. Interestingly, 30% of infected humans will develop cardiomyopathy, digestive implications or both (Rassi Jr and Marin-Neto, 2010), and a small percentage of them may still develop neurological symptoms (Prata, 2001). Although the mechanisms and factors influencing this clinical unpredictability have not been fully elucidated, the variability in the course of Chagas disease seems to be related to a number of factors such as parasite strain, host age, reinfection, and genetic factors of both host and parasite (Prata, 2001).

Since 2009, *T. cruzi* strains have been divided into six discrete taxonomic units, namely *T. cruzi* I – VI, based on its intra-specific genetic variability (Zingales *et al.*, 2009; Baptista *et al.*, 2014). Unquestionably, diverse tissue tropisms, response against immune system, and responsiveness to chemotherapy have been frequently observed in Chagas disease (Revollo *et al.*, 1998; Andrade *et al.*, 2010). In fact, genetic factors are able to strictly regulate infection capacity of parasites, as there is a correlation between genetic diversity and rate of success in escaping the host immune response (Frasch, 2000; Burgos *et al.*, 2013).

It has long been known that several microorganisms display intrinsic, spontaneous mutability events that lead to intra-specific genetic diversity (Steinberg *et al.*, 1971; Taddei *et al.*, 1997; Rosche and Foster, 2000). The generation of spontaneous mutation is a very complex subject since several intrinsic and extrinsic factors might be involved in the process – like the environment in which the organism is found (Matic *et al.*, 1997), location of mutation-prone sites in the genome (Patrushev and Minkevich, 2008), and the behavior of the DNA repair system (Hoeijmakers, 2001). However, a number of studies have already shed light on the mechanisms and importance of spontaneous mutation rate in bacteria (Choi *et al.*, 2011; Ford *et al.*, 2013), yeast (Magni and von Borstel, 1962; Glassner *et al.*, 1998; Bensasson, 2011), and in other non-disease causing eukaryotes (Provan *et al.*, 1999; Shikazono *et al.*, 2003). Also, it has already been shown that certain *T. cruzi* haplogroups display mutations in microssatelite alleles after being cultured in media supplemented with hydrogen peroxide (H_2_O_2_) (Augusto-Pinto *et al.*, 2003).

Therefore, the study of the mechanisms related to the generation of genetic mutations and diversity in *T. cruzi* is imperative since they may play a role in how this parasite deals with genotoxic stress and drug response; in fact, experimental analysis of the antigenic diversity generation remains a challenge since few works tried to investigate *T. cruzi*‘s mutation rate. In this work, we developed a model that allows the detection of mutational events through the selection of *T. cruzi* resistant to the aminoglycoside G418. We found that the mutation frequency in this parasite is similar to other eukaryotic cells, being substantially increased by challenging *T. cruzi* with exogenous H_2_O_2_. Since *T. cruzi* has to cope with oxidative stress situations during its complex life cycle (Piacenza *et al.*, 2009; Machado-Silva *et al.*, 2016), we hypothesize that immunologic evasion and chemotherapy resistance in Chagas disease could be associated to the generation of genetic variability in *T. cruzi* enhanced by oxidative stress conditions.

## Material and Methods

### Plasmid construction and bacterial transformation

Wild-type Neo (Neo^WT^) and its mutant variants – Neo^Δ90^, Neo^Δ180^, Neo^Δ270^, Neo^stop^, and Neo^stopT^→G – were amplified by PCR from the pROCK_Neo vector (da Rocha *et al.*, 2004), using the primers indicated in Table 1. All resultant amplicons (Table 1) were digested with *Xho*I and *Xba*I and then ligated to pMAL-c2G (New England Biolabs Inc., Massachusetts, USA) previously digested with the same endonucleases. Electrocompetent *Escherichia coli* DH5α (Gonzales *et al.*, 2013) were transformed with ligation products and plated onto 2xYT medium [1.6% tryptone, 1.0% yeast extract, 0.5% NaCl (pH 7.0)] supplemented with 100 μg/mL ampicillin. Bacterial positive clones were screened using the colony PCR method (Bergkessel and Guthrie, 2013) and further isolated.

**Table 1:** Primers used for reporter construction.

Primer #	Name	Sequence
**1**	Neo^WT^_FW	ATGGGATCGGCCATTGAACA
**2**	Neo^Δ90^_FW	ATGACAATCGGCTGCTCTGATGC
**3**	Neo^Δ180^_FW	ATGAATGAACTGCAGGACGAGGC
**4**	Neo^Δ270^_FW	ATGGGAAGGGACTGGCTGCTATT
**5**	Neo^stop^_FW	ATGTGATCGGCCATTGAA
**6**	Neo^stopT^→G_FW	ATGGAACAAGATGGATTGCA
**7**	Neo^all^_RV	TCAGAAGAACTCGTCAAG
**8**	Neo^Seq^_RV	ACAGGTCGGTCTTGACA

### Bacterial kanamycin resistance assay

DH5α positive clones for all Neo constructs (Table 1) were grown in 2xYT liquid medium supplemented with 100 μg/mL ampicillin, under orbital agitation (180 rpm) at 37 °C for 16 h. Bacterial cells were then subject of a serial dilution (suspensions with final concentrations of 10^-4^, 10^-6^, 10^-8^, and 10^-10^ cells/mL), and 2.5 μL of each suspension were added onto plates containing 2xYT solid medium (liquid 2xYT plus 2.0% agar) supplemented with 100 μg/mL ampicillin and 0.1 mM isopropyl β-D-1-thiogalactopyranoside (IPTG) in the presence or absence of either 10 μg/mL kanamycin or 10 μg/mL neomycin. Plates were incubated at 37 °C for 18 h at the end of which they were photo-documented.

### *T. cruzi* transfection, selection, and genotyping of transfected clones

Epimastigotes of *T. cruzi* clone CL Brener were grown in liver infusion tryptose medium [0.9% liver infusion broth, 0.5% tryptose, 0.1% NaCl, 0.8% Na_2_HPO_4_, 0.04% KCl, 0.2% hemin, 10% fetal bovine serum; 200 μg/mL streptomycin; 200 μg/mL penicillin (LIT); pH 7.2], at 28 °C. Cells were transfected by electroporation, as described elsewhere (da Rocha *et al.*, 2004), using the pROCK_Hygro-Neo^stop^ construct generated as described in Results, Item 2, and then selected in liquid LIT medium supplemented with 200 μg/mL hygromycin B – cells were transferred to fresh hygromycin B-added LIT weekly, for 4-5 weeks. Then, exponentially-grown transfected cells were plated onto blood-agar medium [48.4% LIT, 48.4% brain-heart infusion and 2.5% defibrinated blood (Gomes *et al.*, 1991)] supplemented with 200 μg/mL hygromycin B for selection of transfected clones. Colony forming units (CFU) were then picked and cultured in hygromycin-added liquid LIT medium, and after 7 days were subjected to genomic DNA extraction as follows: 10^8^ from each *T. cruzi* culture was centrifuged at 5000 x *g* for 5 min and pelleted cells were resuspended in 100 μL Milli-Q water and incubated at 95 °C for 10 min. After another centrifugation, the supernatants were collected and genotyping was conducted by PCR using primers 5 and 8, listed in Table 1.

### Determination of *T. cruzi* growth rate and survival

A defined number (5x10^6^/mL) of transfected *T. cruzi* cells (*T. cruzi*^Neostop^) were cultured for 2 days in fresh hygromycin B-added LIT, until they reached logarithmic growth phase, with cellular concentration around 2x10^7^/mL. After repeating this procedure three times, *T. cruzi*^Neostop^ cells had their growth rate monitored for 7 or 42 days. After that, transfected cells were transferred to hygromycin B-added LIT supplemented with either 200 or 400 μg/mL G418 and cultured for 2 days. The number of viable cells was determined using a hemocytometry chamber by the use of erythrosine as a vital stain for differentiation between live and dead cells. All experiments were performed in biological triplicates and results are reported in mean ± standard deviation. Statistical analyses (one-way ANOVA) were performed using GraphPad Prism v6.0 (GraphPad Software, Inc.).

### *T. cruzi* genomic DNA extraction

*T. cruzi* genomic DNA was extracted through cellular lysis, deproteination and precipitation, as described in Andrade *et al.* (1999). Briefly, a defined number of exponentially-grown *T. cruzi* cells (10^8^) were washed three times with PBS and incubated in 200 μL of lysis solution [(0.5% SDS, 100 μM EDTA, and 10 mM Tris-HCl (pH 8.0)] with 20 μg/mL RNase, for 1 h, at 37 °C. Then, 100 μg/mL proteinase K was added to the lysate, which was incubated at 50 °C for 3 h. Deproteination was conducted by the addition of 200 μL saturated phenol followed by gentle homogenization and centrifugation; the organic phase was then dispose – the same procedures were repeated for the addition of 200 μL of phenol/chloroform 1:1 (v/v) and 200 μL of chloroform. DNA precipitation was carried out using absolute isopropanol at -80 °C overnight. The isopropanolic suspension of DNA was then centrifuged at 16,000 x *g*, for 10 min, and pelleted DNA was washed twice with ethanol 70% before being dried and resuspended in sterile MilliQ water.

### *T. cruzi* genomic DNA sequencing

Genomic DNA from *T. cruzi* was sequenced through the Sanger method using a MegaBACE 1000 DNA Sequencing System (GE Healthcare). For each reaction, DYEnamic ET Dye Terminator MegaBACE kit and the specific set of primers were used. Sequences were analyzed by the Phred-Phrap algorithm (Ewing *et al.*, 1998) and examined with MultAlin for multiple sequence alignment (Corpet, 1988).

### Mutation frequency assay

A defined number of *T. cruzi*^Neostop^ epimastigotes (10^7^) was cultured for 42 days in hygromycin-added LIT in the presence or absence of 50 μM H_2_O_2_. Cells were then washed and resuspended in PBS, and counted as described in Materials and Methods, item 4. A volume of suspension containing 10^8^ cells were plated onto hygromycin B-added solid blood-agar, either in the presence or absence of G418. After 8 weeks, CFUs were counted, and mutation frequency was determined by dividing the number of CFUs observed on the plate per the number of cells/mL present in the liquid LIT culture from which epimastigotes were collected.

## Results

### Development of the Neo^stop^ reporter

We developed a methodology to assess DNA mutation rates in *T. cruzi* based on a system that carries a Neo gene variant unable to encode an amino 3’-glycosyl phosphotransferase [APH(3’)-II] that displays its biological activity, unless a genomic mutational event takes place and restores this ability. APH(3’)-II is an enzyme responsible for microbial resistance against aminoglycosides such as neomycin, kanamycin, and G418 (Hächler *et al.*, 1996).

**Figure 1:**
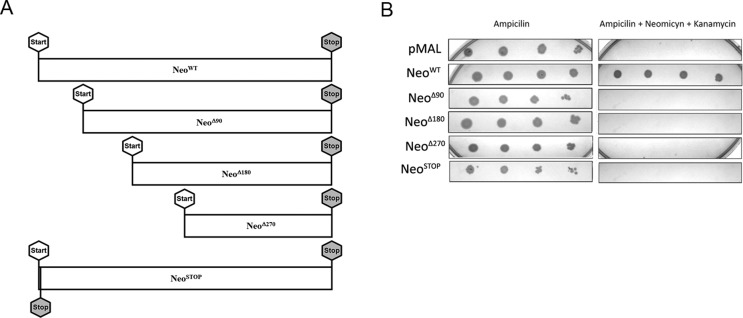
The N-terminal region of Neo is required to promote resistance against kanamycin. Wild-type Neo gene (Neo^WT^) and its variants (Neo^Δ90^, Neo^Δ180^, Neo^Δ270^ and Neo^STOP^) were obtained as described in Materials and Methods, item 1, and kanamycin- and neomycin-resistance assay was conducted as described in Materials and Methods, item 2. (A) Diagram depicting wild-type Neo gene and deletions of N-terminal segments, which give rise to Neo gene variants. (B) Neo^Δ90^, Neo^Δ180^, Neo^Δ270^ and Neo^STOP^ were unable to confer to DH5α resistance against kanamycin. pMAL: empty vector.

First, we sought to determine which segments from APH(3’)-II were essential to its activity. For such, we generated three Neo gene shorter variants lacking their first 90, 180 and 270 nucleotides, using primers 2 – 4, indicated in Table 1. Each Neo gene variant were ligated into pMAL c-2G (which harbors the *lac* promoter; Walker *et al.*, 2010), giving rise to Neo^Δ90^-pMAL, Neo^Δ180^-pMAL and Neo^Δ270^-pMAL constructs (Figure 1A). We next transformed *E. coli* DH5α with all aforementioned constructs, as well as with the wild type Neo construct (Neo^WT^-pMAL) (Figure 1A), and bacterial transformants were selected from 2xYT plates supplemented with 100 μg/mL ampicillin. DH5α transformants were cultivated overnight in liquid ampicillin-added 2xYT, and then plated onto ampicillin-added solid 2xYT supplemented with 0.1 mM IPTG, in the presence or absence of 10 μg/mL kanamycin. We then verified that, unlike Neo^WT^, none of the three obtained Neo gene variants (Neo^Δ90^, Neo^Δ180^, and Neo^Δ270^) were able to confer DH5α resistance against kanamycin (Figure 1A, B). We therefore concluded that the first 30 amino acids of the N-terminal portion of APH(3’)-II are essential to its biological activity.

**Figure 2:**
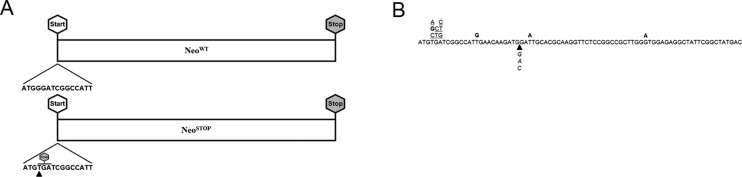
Construction of the Neo^stop^ reporter and manually predicted mutations within its first seventy nucleotides. (A) The Neo^stop^ reporter was constructed substituting a guanine for a thymine at position 4 (as indicated by the arrow), generating the stop codon TGA right after the start codon ATG, as described in *Materials and Methods*, item 1. (B) Manually predicted spontaneous and oxidation-induced mutations within the first seventy nucleotides of the Neo^stop^ reporter are indicated by underlined and bold-type letters, respectively. Insertion of a guanine, cytosine, and adenine at position 26 (indicated by italicized letters) can convert the ATG sequence found at positions 23-25 into an in-frame start codon.

Once we determined that the Neo gene is required to promote resistance against aminoglycosides, we decided to introduce a premature stop codon right after the Neo^WT^ gene start codon using primers 5 and 7 listed in Table 1, creating the Neo^stop^ variant, in which a G – its fourth base – is substituted by a T, generating the stop codon TGA (Figure 2A). This premature stop codon prevents the formation of APH(3’)-II, completely abrogating the growth capacity of DH5α in the presence of kanamycin (Figure 1B). We next manually performed an *in silico* prediction of possible mutations that would restore the translation of the N-terminal portion of APH(3’)-II, and thus provide resistance against aminoglycosides. Interestingly, from all predicted mutational events (Figure 2B), two of them – G→T at position 5, and T→G at position 15 – are classic mutations generated by cellular exposure to H_2_O_2_ (Shibutani *et al.*, 1991).

### Long-term cultivation induces mutational events in *T. cruzi*

**Figure 3:**
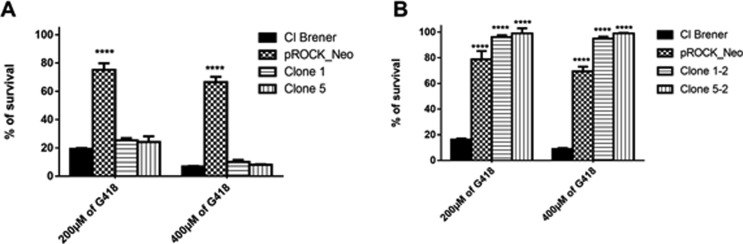
Long term incubation leads to selection of *T. cruz*i^Neostop^ revertant clones. *T. cruzi*^neostop^ transfection, selection, genotyping, and growth rate and survival were determined as described in *Materials and Methods*, items 3 and 4. Panel A: Resistance of *T. cruzi*^Neostop^ epimastigotes against G418 after 7 days of cellular growth in liquid medium. (B) Resistance of *T. cruzi*^Neostop^ epimastigotes against G418 after 42 days of growth in liquid medium, indicating that cell duplication allows mutational events in *T. cruzi*. Epimastigotes harboring pROCK_Neo construct were used as positive controls for aminoglycoside resistance. Statistical analyses (One-Way ANOVA) were conducted using GraphPad Prism software v6.0. ****p < 0.001 vs. WT.

After (i) observing that DH5α transformed with the Neo^stop^-pMAL construct did not exhibit growth in 2xYT supplemented with kanamycin (Figure 1B), and (ii) that oxidation could lead to mutational events that might restore the translation of APH(3’)-II from the Neo^stop^ variant (Figure 2B), we sought to transfect *T. cruzi* with the Neo^stop^ gene variant. As expected, we were unable to observe, through erythrosine vital stain assay, visible growth of clones #1 and #5 of *T. cruzi*^Neostop^ cultured in hygromycin-added liquid LIT supplemented with G418 (Figure 3A). We then investigate if long-term cultivation – *i.e.*, 42 days – of *T. cruzi*^Neostop^ was capable of generating G418-insensitive clones for such, clones #1 and #5 were subject to the same experimental design described above, being cultured for 42 days, instead. Surprisingly, upon the increase of the cultivation period, we were able to verify the presence of G418-resistant *T. cruzi* cells from Neo^Stop^ clones #1 and #5 in hygromycin-added liquid LIT supplemented with 200 or 400 mM G418 (Figure 3B).

### Oxidative stress increases mutational events in *T. cruzi*

Given the fact that long-term cultivation allows the observation of mutational events in *T. cruzi*, we decided to take advantage of the established protocol for isolation of *T. cruzi* clones using solid blood-agar to determine the number of CFUs of G418-insensitive *T. cruzi*^Neostop^ generated from a defined number of plated cells – this would allow us to determine the frequency of mutation of *T. cruzi*. Then, 1x10^8^ cells from Neo^Stop^ clones #1 and #5, previously cultured in hygromycin B-added liquid LIT for 42 days, in the presence or absence of 50 μM H_2_O_2_, were plated onto hygromycin B-added solid blood-agar, and the number of CFUs were determined, as described in Material and Methods, item 7. We verified that *T. cruzi*^Neostop^ cultured in the presence of H_2_O_2_, showed a mutation frequency of 1.56x10^-7^, while parasites cultured in control conditions, *i.e.*, in the absence of H_2_O_2_, exhibited a mutation frequency of 0.71x10^-7^. This observation indicated that there is a two-fold increase in mutation frequency when *T. cruzi* faces situations of environmental oxidative stress. Besides, the experimental design was sensitive enough to allow us to identify the basal frequency of genomic mutations of *T. cruzi*^Neostop^, *i.e.*, the frequency of mutational events observed in parasites that were not exposed to H_2_O_2_ during this assay. This basal frequency – lower than the one observed in the presence of H_2_O_2_ – may indicate the rate of oxidation-independent mutational events that probably take place spontaneously in *T. cruzi*.

### Screening genetic mutations from G418-resistant *T. cruzi*^Neostop^

**Figure 4:**
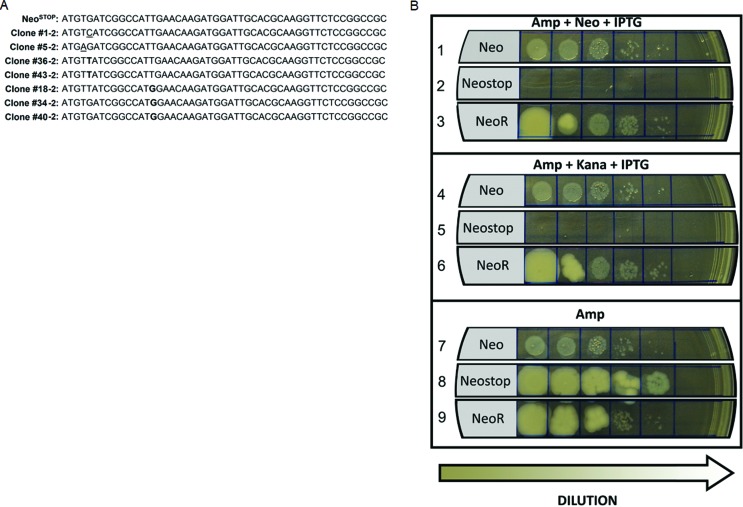
Neo^stopT^→G transversion can rescue aminoglycoside resistance to DH5α. (A) Sequencing analysis of G418-resistant clones shows that exposure to H_2_O_2_ leads to classic transversions arisen from oxidative damage (bold-type letters). Oxidative-unrelated mutations were also found (underlined letters). (B) To verify if T→G at position 15 could restore aminoglycoside resistance in DH5α we generated this transversion through the use of the primer Neo^stopT^→G_FW (#6, Table 1) – which generates a start codon into the Neo^stop^ – to obtain the Neo^stopT^→G reporter, that confers kanamycin- and neomycin-resistance to DH5α.

To determine the identity of the mutations present in G418-resistant *T. cruzi*^Neostop^ clones generated after 42 days of culture in the presence or absence of H_2_O_2_ (Material and Methods, item 7), we next selected seven of them (#1-2 and #5-2, from cultures conducted in the absence of H_2_O_2_; #18-2, #34-2, #36-2, #40-2 and #43-2, from cultures carried out in the presence of H_2_O_2_) aiming to isolate, extract, and sequence their genomic DNA by the Sanger method. Through this screening we verified that (i) *T. cruzi*^Neostop^ clones #1-2, #5-2, #36-2, and #43-2 presented mutations that abrogate the TGA stop codon previously inserted in Neo^stop^ [#1-2: G→A transversion, probably promoted by replicative stress; #5-2: G→C transversion; #36-2 and #43-2: G →T transversions, generated by a 8-oxoguanine (8-oxoG) formed by the oxidation of a guanine from the genomic DNA]; and that (ii) clones #18-2, #34-2 and #40-2 showed a T→G transversion – probably caused by 8-oxoG formation by the oxidation of a guanine from the nucleotide pool at positon 15, allowing the creation of an in-frame start codon at position 13 (Figure 4A). It is noteworthy that all G418-resistant *T. cruzi*^Neostop^ clones picked from the 42-day cellular culture under oxidative stress conditions (#18-2, #34-2, #36-2, #40-2 and #43-2) presented classic transversions that arise from the exposure to reactive oxygen species (Figure 4A). Also, clones #1-2 and #5-2, selected from non-oxidative cellular cultures, despite presenting mutations that abrogate the inserted stop-codon, lacked the classic mutation signature promoted by conditions of oxidative stress.

### The Neo^stopT^→G reporter confers kanamycin resistance to DH5α

We next designed a forward primer carrying a guanine in its 4^th^ position (#6, Table 1) to artificially obtain the Neo^stop^ gene variant mimicking the oxidation-induced T→G mutation, which creates a downstream start codon, as found in Neo^stop^ clones #18-2, #34-2 and #40-2 (Figure 4A). The resultant amplicon (Neo^stopT^→G) was ligated into pMAL c-2G plasmid, generating the Neo^stopT^→G-pMAL construct, which was used to transform DH5α, whose transformants were selected from ampicillin-supplemented 2xYT plates. After isolation, the Neo^stopT^→G-pMAL construct was used to obtain DH5α transformants from solid ampicillin-added 2xYT plates. Once selected, one clone from these bacterial transformants was cultured overnight in liquid ampicillin-added 2xYT, and then plated onto ampicillin-added solid 2xYT supplemented with 0.1 mM IPTG, in the presence or absence of 10 μg/mL kanamycin. We were then able to verify that DH5α harboring Neo^stopT^→G became G418-resistant (Figure 4B), confirming that the aminoglycoside resistance observed in the *T. cruzi*^Neostop^ clones #18-2, #34-2 and #40-2 is in fact promoted by the T→G mutation, a nucleotide transversion classically induced by oxidants (Shibutani *et al.*, 1991).

## Discussion

Genetic diversity is an important factor that is directly related to adaptation and survival of *T. cruzi* in its hosts; in fact, DNA metabolism and mutagenesis may allow this parasite to increase the chances to adapt to different environments during its complex life cycle (Machado-Silva *et al.*, 2016). In this sense, the study of mechanisms that govern this phenomenon is crucial for the understanding of how *T. cruzi* evade the immune system and show resistance against drugs, and for the development of new therapeutic strategies. However, currently, other than a restricted number of studies employing *in silico* approaches to study mutagenesis and variability in *T. cruzi* (Azuaje *et al.*, 2007a,b), there is scarce information regarding the exact cellular events that may generate intra-specific genomic variability and few biological assays that allow the determination and detection of mutation rates in this parasite.

The Neo gene encodes APH(3’)-II, a phosphotransferase that contains 267 amino acids, and is responsible for conferring microbial resistance against aminoglycosides (Hächler *et al.*, 1996). APH(3’)-II displays an ATP biding-site and can transfer the γ-phosphoryl group from an ATP molecule to the aminoglycoside, converting the latter to its phosphorylated, inactive form (Eustice and Wilhelm, 1984; Shaw *et al.*, 1993; Thompson *et al.*, 2002). We generated a number of mutations in the Neo gene, which gave rise to shorter APH(3’)-II variant forms (Table, 1, Figure 1A) that were ineffective in conferring DH5α resistance against kanamycin (Figure 1B). Then, once we determined that the N-terminal segment of Neo was required to provide resistance against kanamycin and G418, we introduced a premature stop-codon right after Neo’s ATG through a G→T mutation at position 4 (Figure 2A), creating a variant (Neo^stop^) that would re-establish resistance against aminoglycosides if the mutated codon underwent a mutational event. This was observed when *T. cruzi*^Neostop^ was cultivated for 42 days in hygromycin B-added liquid LIT (Figure 3).

During its life cycle, *T. cruzi* undergoes an obligatory intracellular amastigote stage in which the immune system promotes the release of reactive oxygen and nitrogen species to halt the infection (Piacenza *et al.*, 2009); thus, replication of amastigotes under a scenario of oxidative stress can promote a condition from which mutated cells can ultimately increase the pool of mutated *T. cruzi*, which could lead to intra-specific genetic diversity. Although epimastigotes and amastigotes are subjected to different extents of oxidative stress, data from the literature (Aguiar *et al.*, 2013), as well as unpublished observations from our group, suggest that both aforementioned *T. cruzi* life forms are equally affected by oxidative stress and share the same responses against this biological condition. Therefore, the observation that epimastigotes treated with H_2_O_2_ display a 2-fold increase in mutational events (Results, item 3) suggests that oxidative stress promoted by the host may play a direct role in genetic variability of *T. cruzi* amastigotes. In fact, for several other organisms, including *E. coli*, *Helicobacter pylori*, *Salmonella typhimurium*, *Bacillus subtilis*, *Pseudomonas*, *Clostridium*, *Saccharomyces cerevisiae*, and *Candida albican*s, increased mutation rates are often correlated with increased survival and infection rates in adverse conditions (Wang *et al.*, 2001; Foster, 2000; Linz *et al.*, 2014). In this manner, the increase in the number of G418-resistant *T. cruzi*^Neostop^ clones after long-term oxidative insult (Figure 3) suggests that this type of stress could stimulate intra-specific genetic variability.

It is well-established that oxidative stress promotes a range of modifications in nucleic acids, such as double-strand breaks and nitrogenous base modification (Friedberg *et al.*, 2006). Interestingly, the generation of 8-oxoG, one of the most frequent lesions derived from oxidative stress, has a high mutagenic potential, since the oxidized guanine, if localized in the genomic DNA, promotes a mismatched pairing with adenine resulting in G→T or C→A transversions. In addition, the generation of 8-oxoG in the nucleotide pool also promotes a T→G transversion, consequently leading to nucleotide mismatches (Dizdaroglu *et al.*, 2002; van Loon *et al.*, 2010). In fact, the severity of effects that can arise from the formation of 8-oxoG became evident when the GO system – a pathway specialized in preventing mutagenicity promoted by 8-oxoG, comprised of three enzymes, namely MYH (MutY homologue), MTH (MutT homologue), and OGG1 (FPG homologue) – was first described (Michaels *et al.*, 1992; Michaels and Miller, 1992; David *et al.*, 2007).

In *T. cruzi*, long-term exposure to H_2_O_2_ induced DNA mutations related to the generation of 8-oxoG, as clones #18-2, #34-2 and #40-2 showed mutations that are likely consequence of a guanine oxidation (Figure 4A). Likewise, clones #36-2 and #43-2 also presented formation of 8-oxoG mutations, since guanine in DNA undergoes a mispairment with adenine during replication (Figure 4A). The mispairing observed in clones #1-2 and #5-2 – which were not exposed to H_2_O_2_ – are possibly products of an impaired replication process induced by a wobble conformation, although the DNA template and protein conformation are not disturbed (Johnson and Beese, 2004). These mismatches allow the formation of a structure closer to Watson-Crick base pair than that one observed in G:A and A:G mismatches. Altogether, these verifications indicate that mutations observed in *T. cruzi* cells exposed to H_2_O_2_ are products of generation or misincorporation of 8-oxoG in the DNA, since those mutations are deleterious and do not easily arise in normal environments, considering the abnormalities they cause to the polymerase structure (Johnson and Beese, 2004). Alterations in DNA metabolism can also increase genetic mutation frequency (Castillo-Acosta *et al.*, 2012). Organisms like yeast seem to preferentially insert cytosine opposing apurinic/apyrimidinic sites, and this mechanism could lead to the increase of AT→GC transversions (Thomas *et al.*, 1997).

As suggested for *T. cruzi,* the presence of mutations, to some extent, are possibly related to the survival of some other organisms. In fact, *Trypanosoma brucei* strain relies on variant surface glycoproteins (VSG) switching to escape from the host immune system, a process in which recombination plays a crucial role (Hartley and McCulloch, 2008; Horn and McCulloch, 2010). Deletion of deoxyuridin 5’-triphosphate pyrophosphatase (dUTPase) can cause a 9-fold increase in spontaneous mutation, and the appearance of double strand breaks in *T. brucei*, which could lead to a recombination process, increasing VSG switching (Castillo-Acosta *et al.*, 2012).

In this work, through a novel assay to assess mutational events in *T. cruzi*, we demonstrated that oxidative stress increases the mutation frequency in this parasite. We hypothesize that the 2-fold increase in mutation frequency after exposure to H_2_O_2_ – which mimics the reactive oxygen species released by human macrophages – indicates that this mutational mechanism, combined with the GO repair system – could generate *T. cruzi*‘s intra-specific genetic diversity that can be important to help this trypanosomatid to evade the immune system and be resistant to drug therapy, ultimately allowing this parasite to survive in stressful environments.
